# Modelling of the cathodic and anodic photocurrents from *Rhodobacter sphaeroides* reaction centres immobilized on titanium dioxide

**DOI:** 10.1007/s11120-018-0550-8

**Published:** 2018-07-03

**Authors:** Rafał Białek, David J. K. Swainsbury, Maciej Wiesner, Michael R. Jones, Krzysztof Gibasiewicz

**Affiliations:** 1 Faculty of Physics, Adam Mickiewicz University in Poznań, ul. Umultowska 85, 61-614 Poznan, Poland; 2 School of Biochemistry, Biomedical Sciences Building, University of Bristol, University Walk, Bristol, BS8 1TD UK; 3 NanoBioMedical Center, Adam Mickiewicz University in Poznań, ul. Umultowska 85, 61-614 Poznan, Poland; 4 Present Address: Department of Molecular Biology and Biotechnology, University of Sheffield, Sheffield, S10 2TN UK

**Keywords:** Photovoltaics, Purple bacteria, Bioelectronics, Titanium dioxide, Photosynthesis

## Abstract

As one of a number of new technologies for the harnessing of solar energy, there is interest in the development of photoelectrochemical cells based on reaction centres (RCs) from photosynthetic organisms such as the bacterium *Rhodobacter* (*Rba*.) *sphaeroides*. The cell architecture explored in this report is similar to that of a dye-sensitized solar cell but with delivery of electrons to a mesoporous layer of TiO_2_ by natural pigment-protein complexes rather than an artificial dye. *Rba. sphaeroides* RCs were bound to the deposited TiO_2_ via an engineered extramembrane peptide tag. Using TMPD (*N,N,N′,N′*-tetramethyl-*p*-phenylenediamine) as an electrolyte, these biohybrid photoactive electrodes produced an output that was the net product of cathodic and anodic photocurrents. To explain the observed photocurrents, a kinetic model is proposed that includes (1) an anodic current attributed to injection of electrons from the triplet state of the RC primary electron donor (P^T^) to the TiO_2_ conduction band, (2) a cathodic current attributed to reduction of the photooxidized RC primary electron donor (P^+^) by surface states of the TiO_2_ and (3) transient cathodic and anodic current spikes due to oxidation/reduction of TMPD/TMPD^+^ at the conductive glass (FTO) substrate. This model explains the origin of the photocurrent spikes that appear in this system after turning illumination on or off, the reason for the appearance of net positive or negative stable photocurrents depending on experimental conditions, and the overall efficiency of the constructed cell. The model may be a used as a guide for improvement of the photocurrent efficiency of the presented system as well as, after appropriate adjustments, other biohybrid photoelectrodes.

**Electronic supplementary material:**

The online version of this article (10.1007/s11120-018-0550-8) contains supplementary material, which is available to authorized users.

## Introduction

**Fig. 1:**
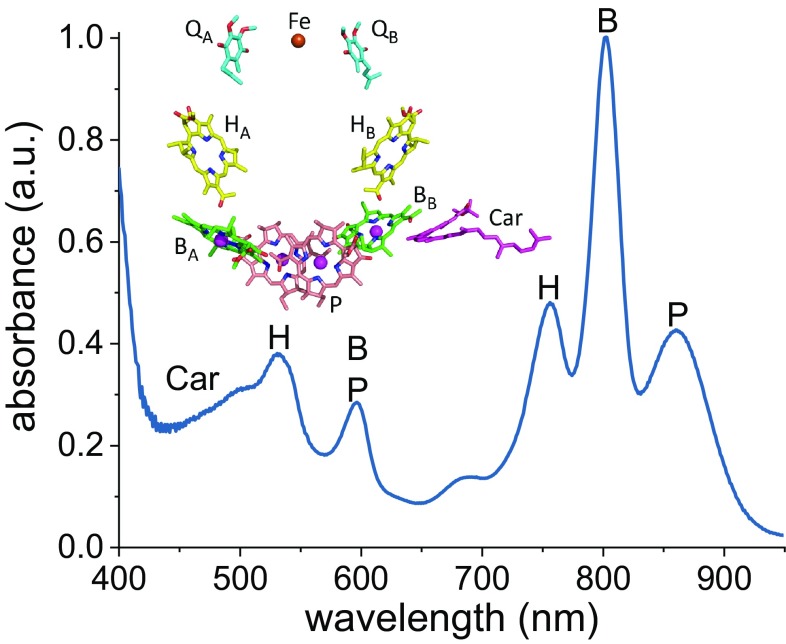
Cofactor structure and absorbance spectrum of the *Rba. sphaeroides* RCs. For the cofactor structure, color coding is cyan, yellow, green, pink or magenta—carbon; red—oxygen; blue—nitrogen; purple spheres—magnesium; brown sphere—iron. Bands in the absorption spectrum of RCs in 20 mM Tris–HCl (pH 8.0)/0.1% LDAO are labelled with the names of the contributing cofactors

Sunlight is arguably the most sustainable source of energy for mankind. Nature has evolved very efficient molecular processes for the conversion of solar energy that have provided inspiration for the design of man-made photovoltaic materials and provide natural components that can be exploited directly in biohybrid devices. One of the best characterized of these is the reaction centre (RC) from the purple photosynthetic bacterium *Rhodobacter* (*Rba*.) *sphaeroides*, a complex of protein and cofactors in which photon absorption powers charge separation (Zinth and Wachtveitl 2005). The protein provides a matrix that holds in place two primary electron donor (P) bacteriochlorophylls (BChls), two accessory BChls (B_A_ and B_B_), two bacteriopheophytins (BPhe - H_A_ and H_B_), two ubiquinones (Q_A_ and Q_B_) and a carotenoid (Car) (see inset in Fig. 1) (D’Haene et al. 2014). The initial charge separation occurs between the P BChls and one of the two BPhes, forming the state P^+^H_A_^−^. Subsequently, there are three ways electron transfer can proceed. In “open” RCs, with all of the electron transfer cofactors initially in their neutral ground state, the electron is transferred to the first of the ubiquinones, Q_A_, and then on to the second, Q_B_, completing photochemical charge separation. In “closed” RCs, where Q_A_ is already reduced, the most probable event is recombination of P^+^H_A_^−^ to the ground state. In a smaller percentage of RCs, recombination occurs to a long-lived triplet excited state of P, termed P^T^ (Woodbury and Allen 1995), which is usually efficiently quenched either by the carotenoid or by the BPhes (Arellano et al. 2004; Białek et al. 2016). The quantum yield of primary charge separation in open RCs is near 100% (Wraight and Clayton 1974), while the yield of P^T^ triplet formation in closed RCs is approximately 15% (Blankenship et al. 1995).

One of the most promising alternatives to silicon cells for solar energy conversion is the dye-sensitized solar cell (DSSC) (O’Regan and Grätzel 1991). This consists of a working electrode made of a material such as fluorine-doped tin oxide (FTO) conductive glass coated with a mesoporous TiO_2_ film that is covered with a layer of dye molecules, and a counter electrode also made of conductive glass. Between the electrodes there is a solution containing an electrolyte, originally iodide/triiodide (O’Regan and Grätzel 1991), which closes the electrical circuit inside the cell by allowing electrons to be transported between the two electrodes. The TiO_2_ film provides a three-dimensional semi-conducting matrix which improves light harvesting efficiency by increasing the surface area onto which the sensitizing dye can bind. Photoexcitation of the dye causes charge injection into the conduction band of the TiO_2_, followed by re-reduction of the dye by the electrolyte.

A feature of the ruthenium dyes commonly used in DSSCs is their limited ability to absorb light beyond 700 nm, with many having no significant absorbance beyond 800 nm, regions which are photon-rich in natural sunlight (Nazeeruddin et al. 2011). In contrast, as illustrated in Fig. 1, pigment-proteins from organisms containing BChl *a* have very strong absorbance in the near infrared between 700 and 900 nm, and up to 1100 nm in organisms that contain BChl *b* (Mikhailyuk et al. 2006). Thus, a possible modification of the design of the DSSC is to replace the synthetic dye with a photoactive pigment-protein such as a RC. An additional benefit is that, unlike many synthetic dyes, natural pigment-proteins are not harmful to the environment. Bacterial RCs and other photosynthetic proteins such as Photosystem I (PSI) have been tested in a variety of prototype photovoltaic devices (Lu et al. 2007; Nagy et al. 2010). Substrates employed have typically been flat metal surfaces (Ciesielski et al. 2010; den Hollander et al. 2011; Chen et al. 2013; Swainsbury et al. 2014), or alternatively flat (Tan et al. 2012a, b; Caterino et al. 2015) or porous (Lu et al. 2005b, a; Lukashev et al. 2007; Nadtochenko et al. 2008; Woronowicz et al. 2012; Mershin et al. 2012; Nikandrov et al. 2012; Gizzie et al. 2015b; Shah et al. 2015; Yu et al. 2015; Kavadiya et al. 2016) semiconductor layers. A porous semiconductor film provides an up to 2000-fold higher surface area than that can be achieved with a planar electrode of the same 2-D area (O’Regan and Grätzel 1991) and materials such as TiO_2_ are much cheaper than the precious metals such as gold and platinum commonly used for planar electrodes. In previous work, both PSI (Mershin et al. 2012; Nikandrov et al. 2012; Gizzie et al. 2015b; Shah et al. 2015; Yu et al. 2015; Kavadiya et al. 2016) and the purple bacterial RC (Lu et al. 2005a, b; Lukashev et al. 2007; Nadtochenko et al. 2008; Woronowicz et al. 2012) have been deposited on TiO_2_ porous substrates for the study of photocurrent generation. The highest photocurrents obtained so far for a photosynthetic protein-TiO_2_ composite cell were presented by Shah et al., who achieved current densities of a few hundreds of µA cm^−2^ using PSI and a nanostructured leaf-like TiO_2_ (Shah et al. 2015). A variety of protein deposition methods, electron mediators and formulations of TiO_2_ layer have been explored. However, none of these studies have attempted a full model of electron transport within the cell, with only schematic diagrams of the selected processes that underlie the photocurrent.

In this study, a photoelectrochemical cell based on *Rba. sphaeroides* RCs, TiO_2_, conducting glass and a redox mediator is investigated through a combination of experiment and modelling. To obtain oriented, self-directed binding to the working electrode, the RC was engineered with a TiO_2_-binding peptide exposed at the electron donor side (P-side) of the protein. The usage of these particular materials for working electrode was a way to have mixed anodic and cathodic photocurrents, despite the tag. The net photocurrents obtained from the engineered RCs were either cathodic or anodic, depending on how the TiO_2_ electrode was prepared. To explain the mechanism of photocurrent generation in detail, a series of electrochemical and spectroscopic measurements were conducted and a kinetic model was prepared. This model, which includes electron transfer from the P^T^ state to the TiO_2_, electron transfer from surface states of the TiO_2_ to P^+^, and interactions of the redox mediator with RCs and the conductive electrode surface, explains the principal features of the observed photocurrent transients and reveals the factors that limit the photocurrent outputs of the cells.

## Results and discussion

### Photocurrents from RC working electrodes

**Fig. 2:**
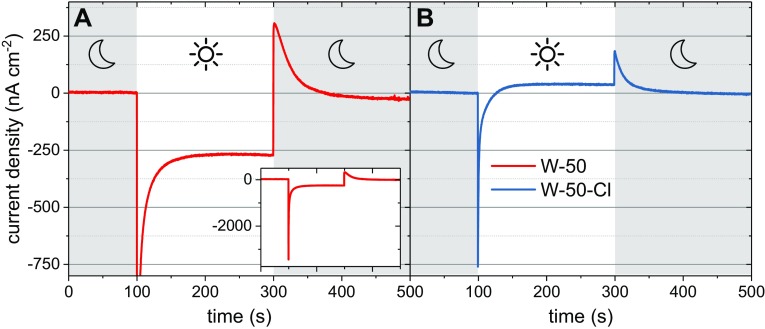
Photocurrent generation by RC/TiO_2_ electrodes in response to illumination at ~ 860 nm. Typical photochronoamperometric data are shown for W-50 electrodes (**a**) without and (**b**) with TiCl_4_ treatment prior to protein adherence. The inset in panel A shows same data over their full amplitude range. Positive currents mean an anodic process. Grey background indicates periods without illumination

**Fig. 3:**
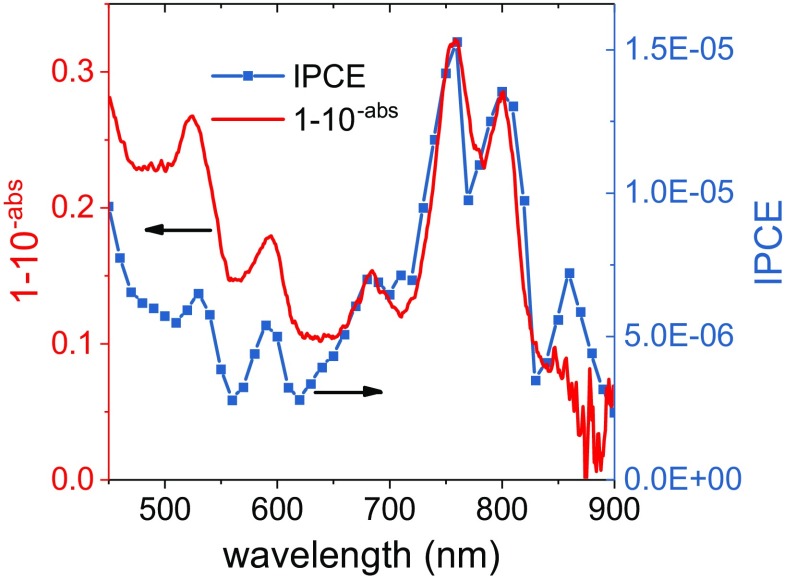
Source of photocurrents. The IPCE action spectrum (blue) and absorptance spectrum (red) for an I-50 RC electrode are compared. Each point of the IPCE spectrum was constructed from the magnitude of the cathodic photocurrent after 10 s of illumination (see "Experimental section" for details)

Protein-coated electrodes submerged in an electrolyte solution comprising 250 µM TMPD in 20 mM tris (pH 8.0) produced photocurrents, an example of which is shown in Fig. 2a for RCs adhered to a W-50 TiO_2_ film (see "Experimental section”). Turning on the light produced a negative (cathodic) peak of current density that decayed nonexponentially to a constant level. Turning off the light produced a positive (anodic) peak followed by a nonexponential decay to around zero current. No photocurrents were obtained when TiO_2_ electrodes without RCs were immersed in the TMPD electrolyte, showing that the photocurrent was dependent on the photochemical activity of the RC. In agreement, an action spectrum of incident photon to current efficiency (IPCE) as a function of excitation wavelength matched the absorbance spectrum of the TiO_2_-bound RCs (Fig. 3) but did not contain contributions from TMPD/TMPD^+^ between 450 and 650 nm (Figure S3), confirming that the photocurrent was being driven by the RC. The absorbance and IPCE action spectra of RCs bound to TiO_2_ (Fig. 3) showed an increase in the absorbance band at 760 nm relative to that at 802 nm which we attribute to partial pheophytinization of RC BChls caused by binding of the protein to TiO_2_ (compare Figs. 1, 3; see also Figure S4 and Sect. 4 and 5 in Supporting Information). The absorption spectra of working electrodes before and after (photo) electrochemical experiments showed no significant differences in line shape (data not shown). This confirmed that no further pheophytinization took place during measurements, and that the protein was stable on the electrode surface when submerged in buffer solution. The maximum measured value of IPCE was 1.5 × 10^−5^ (Fig. 3), which is much lower than efficiencies reported in the literature for systems containing photosynthetic proteins immobilized on nanostructured TiO_2_ (Mershin et al. 2012).

Photocurrents recorded for working electrodes that had been treated using TiCl_4_ (see "Experimental section") showed a different behaviour in which the initial spike of cathodic current decayed to a steady anodic current (Fig. 2b). The size of the light-on and light-off spikes of cathodic and anodic current, respectively, were also strongly affected by TiCl_4_ treatment.

### Mechanism of anodic and cathodic photocurrents

Such photocurrent transients with opposing current spikes on light-on/off have been presented previously in the literature for RC cells not involving TiO_2_ (Tan et al. 2012a; Caterino et al. 2015; Friebe et al. 2016) and explained in different ways. Friebe et al. (2016) attributed this effect to the diffusion-limited transfer of mediator to and from the working electrode and showed that this could be overcome by use of a rotating disc electrode to achieve mixing. A similar explanation was presented by Tan et al. (2012a), who proposed that the bottleneck reaction is reduction of TMPD^+^ by quinone in the RC. This leads to a capacitor-like behaviour, where absorption of light causes accumulation of electrons on cofactors of RC and positive charges in TMPD^+^, with discharge of the system after turning off the light observable as an anodic photocurrent. This explanation may be sufficient for a situation in Fig. 2a, where both the initial light-on spike and steady-state current are cathodic, but does not explain the anodic stable photocurrent illustrated in Fig. 2b.

An alternative explanation for photocurrent transients of this type has been presented by Caterino et al. (2015), based on the concept that both cathodic and anodic photocurrents coexist, but with different kinetics. The source of these two currents was proposed to be interactions of either the P (oxidizing) or Q (reducing) sides of the RC with the electrode. The peak current after light-on arises mostly from the cathodic contribution while the peak after light-off arises mostly from the anodic contribution. The sign of the stable current is determined by the relative magnitudes of the stable cathodic and anodic components.

Taking into account all the abovementioned hypotheses, we build a kinetic model with different sources of anodic and cathodic photocurrents being presented in the following paragraphs.

**Fig. 4:**
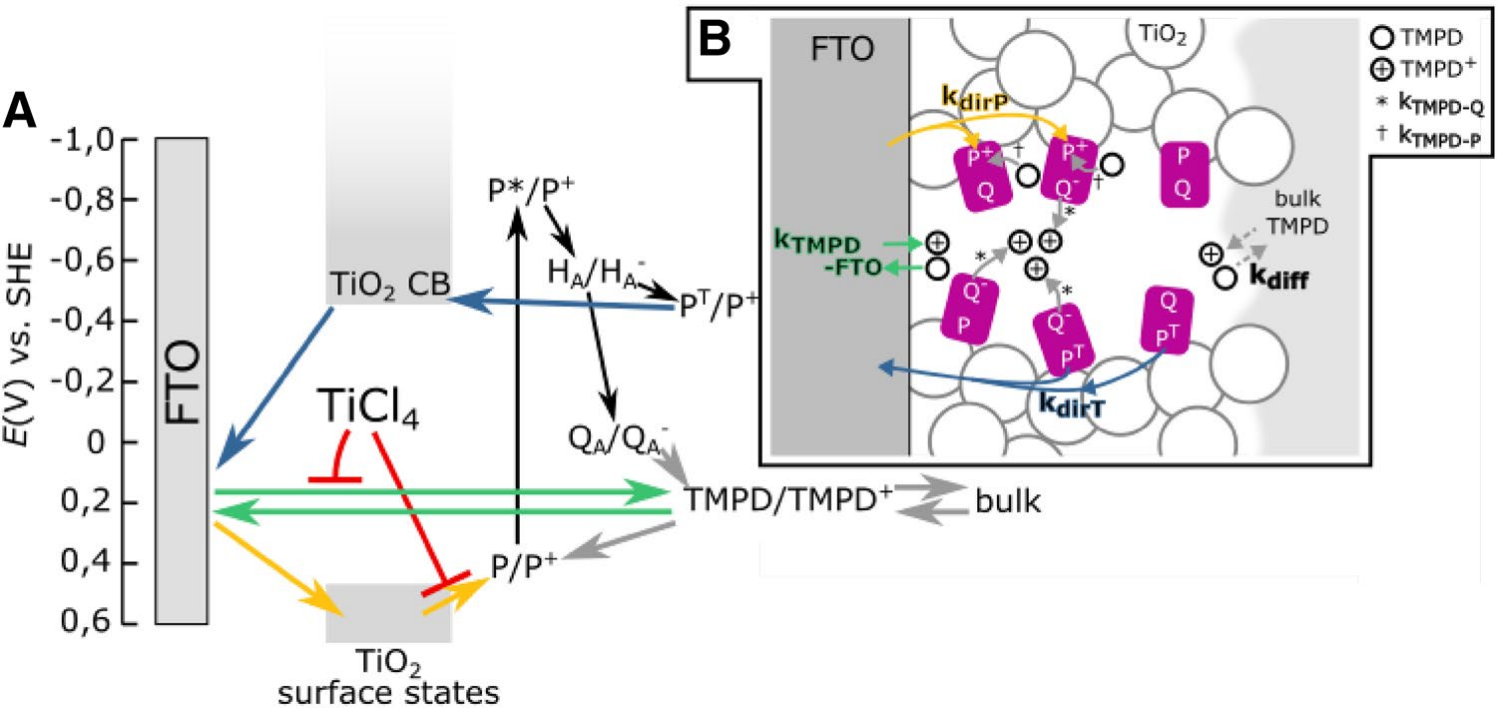
Modelling to account for the mechanism of photocurrent generation and shapes of photocurrent transients. **a** Scheme of energy levels and processes in the system. Green, blue and yellow arrows correspond to three different processes that contribute to the net current. Black arrows—processes occurring inside RCs, red lines—suppression of electron transfer by TiCl_4_ treatment, grey arrows—other electron transfer processes including recombination mediated by TMPD/TMPD^+^. CB signifies conduction band, TMPD/TMPD^+^ signifies the mediator redox pair inside pores, bulk signifies redox mediator within the bulk volume of the electrochemical cell. **b** Scheme of the same processes as in panel A but depicting the architecture of the electrode and charge transfer reactions occurring between FTO, TiO_2_, RCs and mediator inside a TiO_2_ pore. Six RC states are considered: PQ_A_, P^+^Q_A_^−^, P^+^Q_A_, PQ_A_^−^, P^T^Q_A_^−^ and P^T^Q_A_. Four of these states may exchange electrons with TMPD/TMPD^+^: P^+^Q_A_^−^, P^+^Q_A_, PQ_A_^−^ and P^T^Q_A_^−^. Two of the states may inject the electron to TiO_2_: P^T^Q_A_^−^ and P^T^Q_A_. Two of the states may take the electron from TiO_2_: P^+^Q_A_^−^ and P^+^Q_A_. Two of the states may be photoexcited: PQ_A_ and PQ_A_^−^

Regarding the observed anodic steady-state current (Fig. 2b), it has previously been proposed that electrons can be injected into the conduction band of TiO_2_ from the P^T^ triplet excited state of the primary donor BChls (Fig. 4, blue arrows) (Lukashev et al. 2007). P^T^ is usually short-lived in *Rba. sphaeroides* RCs due to transfer of energy to the RC carotenoid (Car) via the intervening B_B_ BChl in around 40 ns (Angerhofer et al. 1998). However, as described in Sect. 4 of Supporting Information, it is likely that a significant fraction of this B_B_ BChl undergoes pheophytinization after deposition of RCs on the TiO_2_ porous layer. It has been shown previously in the literature that genetic replacement of the native B_B_ BChl by a BPhe leads to an increase of the lifetime for triplet energy transfer from P^T^ to the Car to around 1.6 µs (Mandal et al. 2017). This raises the possibility that, in the present work, P^T^ may also have an extended lifetime in a large majority of RCs. Given this, in our proposed model the anodic photocurrent is attributed to electron transfer from P^T^ to the conduction band of TiO_2_ (Fig. 4, blue arrows), with re-reduction of the resulting P^+^ by TMPD. Although it has a suitable reduction potential, electron injection from H_A_^−^ into the conduction band of TiO_2_ is unlikely as this cofactor is deeply buried within the RC and the lifetime of H_A_ is short (~ 200 ps if Q_A_ is neutral and may accept the electron from H_A_^−^ (Woodbury and Allen 1995), and a few ns if Q_A_ is reduced to Q_A_^−^ (Woodbury and Parson 1984; Gibasiewicz and Pajzderska 2008; Gibasiewicz et al. 2011)). The higher energy P* singlet excited state has an even shorter lifetime of ~ 3 ps and decays to P^+^H_A_^−^ with a close to 100% quantum yield. Electron injection from Q_A_^−^ into the conduction band of TiO_2_ is unlikely due to too positive redox midpoint potential of Q_A_^−^/Q_A_ (Fig. 4).

Regarding the cathodic current, TiO_2_ electrodes have surface states that lie between the conduction and valence bands, at around + 550 mV versus SHE (Fig. 4), the value obtained for a set of redox mediators in acetonitrile solution (Frank and Bard 1975). This is slightly above the P/P^+^ redox midpoint potential of the RC primary donor (+ 500 mV versus SHE) (Maróti and Wraight 2008). As it is known that in aqueous basic solutions the conduction band is shifted towards less positive potentials than in organic solvents (Fitzmaurice 1994), there is a possibility that the potential of the TiO_2_ surface states in our system was also shifted toward less positive potentials, making electron transfer from these states to the oxidized RC primary donor (P^+^) more favourable. Thus, we propose that the source of the cathodic photocurrent is the transfer of electrons from the FTO electrode through the surface states of TiO_2_ to P^+^ (Fig. 4, yellow arrows), with TMPD carrying electrons from the RC quinones to the counter electrode. The injection of electrons from any state of the RC into the surface states of TiO_2_ is unlikely due to occupation of these surface states with electrons at the applied potential of + 225 mV versus SHE (Frank and Bard 1975). As with an anodic current dependent on P^T^, this mechanism for the cathodic current would be expected to be facilitated by attachment of the RC to the TiO_2_ by a protein tag that positions the P BChls close to the TiO_2_ surface. However, in both cases, productive electron exchange with the TiO_2_ is expected to be in competition with energy losses through its dissipation within RCs, such that at any given time the photocurrent is supported by only a sub-set of RC proteins where P^T^ oxidation or P^+^ reduction by the adjacent TiO_2_ is possible. There is a possibility that some of the RCs are not properly attached to TiO_2_ (e.g. freely diffusing in pores) which favours inner energy dissipation, thus these RCs could be treated as a source of parasitic absorption.

Regarding the interaction of the mediator with RCs it has been reported that the TMPD/TMPD^+^ redox pair can either reduce P^+^ or oxidize Q^−^ (Fig. 4), with the rate constant for reduction of P^+^ being around 200-fold faster than for the oxidation of Q (Agalidis and Velthuys 1986). However, these results were obtained for RCs and TMPD freely diffusing in solution. In the case of RCs immobilized in the porous TiO_2_ matrix, the two reaction rate constants could be significantly different from solution values. Thus, in one of the models (RMIL—see “Simulation of photocurrents using a kinetic model”) these rate constants were left as free parameters in optimization. In addition to redox interactions with RC cofactors there is the possibility that the TMPD/TMPD^+^ electrolyte can interact with the FTO glass electrode either directly at any locations where the FTO is not fully covered by the TiO_2_ layer, or via tunnelling in any areas where the FTO is covered by only a very thin layer of TiO_2_ from TiCl_4_ treatment (see "Experimental section"). However, the redox potential of TMPD/TMPD^+^ is unsuited to an exchange of electrons with TiO_2_ itself (Fig. 4). As the potential applied to working electrode (+ 225 mV SHE) was close to the midpoint potential of the TMPD/TMPD^+^ couple, in darkness, the [TMPD]/[TMPD^+^] ratio in the vicinity of the working electrode should be around one, similarly as in the bulk solution (see Fig S5 and Sect. 6 and 7 of Supporting Information). However, our modelling shows that under illumination, the local value of this ratio in the immediate vicinity of the mesoporous surface may be transiently or even permanently significantly different from one (see Figs. S7 and S8). Therefore, diffusion of the oxidized and reduced forms of the mediator between the mesoporous region near the electrode surface (pores) and the bulk solution also has to be taken into account.

### Simulation of photocurrents using a kinetic model

A set of differential equations was used to model the experimental data demonstrating a net cathodic stable photocurrent from the W-50 electrodes and a net anodic stable photocurrent from the W-50-Cl electrodes (Fig. 2). A simplified schematic of this model is shown in Fig. 4B, a detailed account of the physical and mathematical basis for the model is given in Fig. S6 and Sect. 8 of Supporting Information. Two sets of conditions were considered (1) only 1 − *x* = 10% of RCs achieve electron transfer between TiO_2_ and the mediator, while 90% of RCs dissipate the energy (a so-called “inactive pool” (IP) model) and (2) all RCs achieve such electron transfer but the rate constants of electron transfer reactions between RC and TMPD/TMPD^+^ are smaller than those cited in the literature [a so-called “RC-mediator interface limited” (RMIL) model]. For both conditions, some parameters were taken from the literature, while others were optimized to achieve the best fit to the experimental photocurrent transients (see Table 1 and Sect. 8 of Supporting Information).

**Table 1:** Simulation parameters for the IP and RMIL models

Parameter	Unit	Value			
		RMIL model no TiCl_4_	RMIL model with TiCl_4_	IP model no TiCl_4_	IP model with TiCl_4_
${k_{{\text{TMPD}} - {\text{P}}}}$	mol^−1^ m^3^ s^−1^	2		800*	
${k_{{\text{TMPD}} - {\text{Q}}}}$	mol^−1^ m^3^ s^−1^	0.6		4*	
$\chi$	–	0.0		0.9	
${k_{{\text{dirT}}}}$	s^−1^	5 × 10^1^		8 × 10^3^	
${k_{{\text{dirP}}}}$	s^−1^	9.7 × 10^−2^	4.5 × 10^−2^	8.0 × 10^3^	3.5 × 10^2^
${k_{TMPD - FTO}}$	m s^−1^	8 × 10^−7^	7 × 10^−7^	1.7 × 10^−7^	1.7 × 10^−7^
${k_{{\text{diff}}}}$	mol^−1^ m^3^ s^− 1^	5.5	8	3.3 × 10^−1^	3.3 × 10^−1^
${k_{{\text{h}}\nu }}$**	mol m^−3^ s^−1^	2.3	2.7	2.0 × 10^−2^	7.2 × 10^−2^
${k_{{\text{h}}\nu {\text{T}}}}$**	mol m^−3^ s^−1^	6.9	6.4	9.1 × 10^−1^	9.0 × 10^−1^

For the meaning of the parameters see main text, Fig. 4, and Fig. S6 in Supporting information

*values taken from the literature and fixed (Agalidis and Velthuys 1986)

**values after 299 s (end of the steady photocurrent phase)

**Fig. 5:**
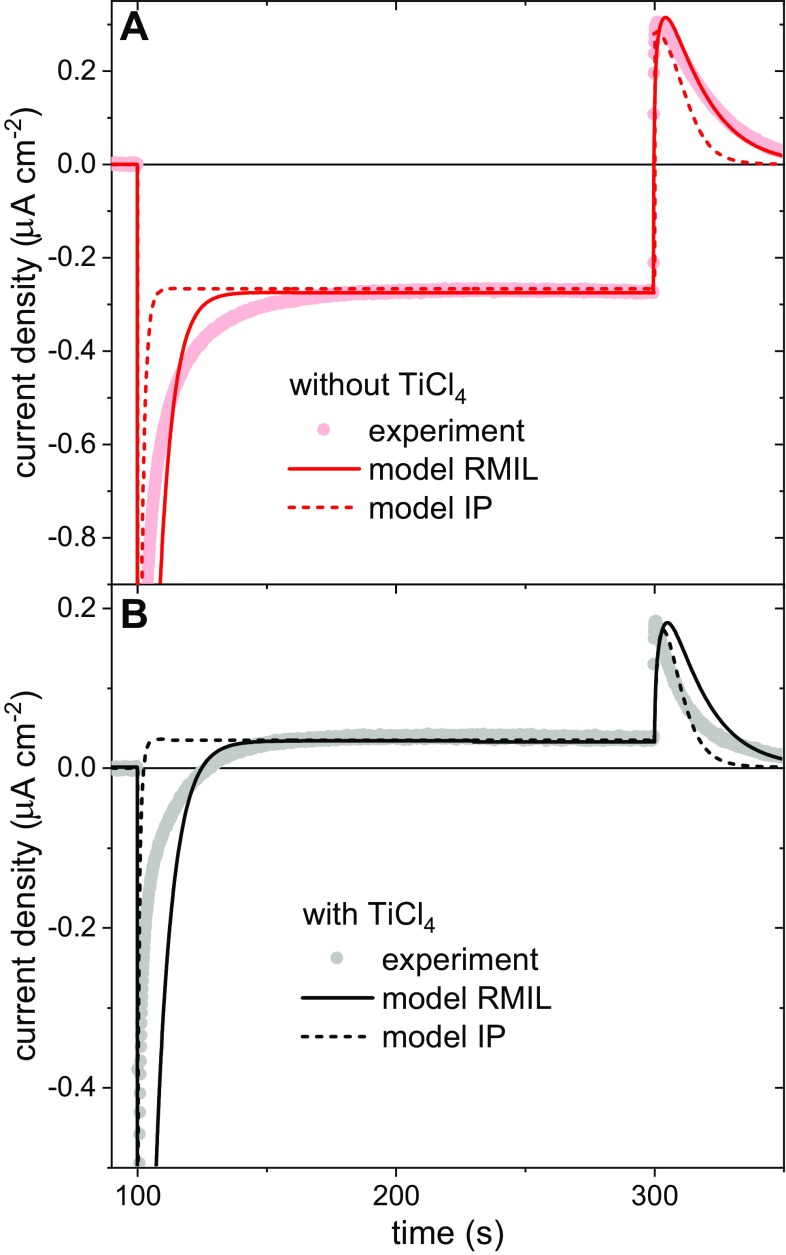
Simulations of photocurrent transients. Simulations based on two models (lines) for electrodes **a** without and **b** with TiCl_4_ treatment are compared with experimental data from Fig. 2 (circles)

The resulting simulated photocurrent transients are shown in Fig. 5, overlaid with the experimental data. Accounting for the difference in the data with and without TiCl_4_ (Fig. 5B versus 5A**)** required variation of only three parameters, the rate constant for electron transfer from TiO_2_ surface states to ${{\text{P}}^+}\,({k_{{\text{dirP}}}})$, the rate constant for electron transfer between FTO and TMPD/TMPD^+^ $({k_{{\text{TMPD}} - {\text{FTO}}}})$, which was the same in both directions and the rate constant for TMPD/TMPD^+^ diffusion $({k_{{\text{diff}}}})$ (Fig. 4b). Table 1 contains resulting values of all the parameters. As it is presented in literature (O’Regan et al. 2007), the TiCl_4_ treatment decreases the number of surface states thus electron transfer from TiO_2_ surface states to P^+^ is suppressed $({k_{{\text{dirP}}}}$ is decreased). This effect is depicted by the red lines in Fig. 4a. On the other hand, the expected suppression of electron transfer between FTO and TMPD/TMPD^+^ by TiCl_4_ treatment was rather limited $({k_{{\text{TMPD}} - {\text{FTO}}}})$, and the value of this parameter was strongly dependent on the value of the third parameter, the rate constant for TMPD/TMPD^+^ diffusion (${k_{{\text{diff}}}}$; these two parameters were compensatory). The values used for these two latter rate constants were chosen to properly model the shape of the spike of positive current obtained after turning off the light. Proper interpretation of these two rate constants will require additional independent experiments to obtain the value of at least one of them.

**Fig. 6:**
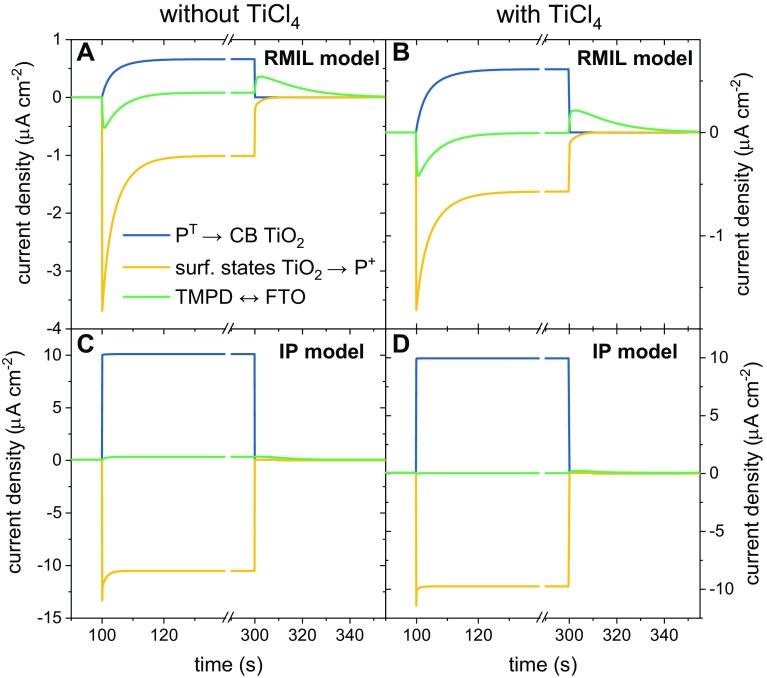
Photocurrent components. Plots present time traces of the three component photocurrents derived from the **a, b** RMIL model with all RCs active and **c, d** IP model with a 90% pool of inactive RCs

The simulated photocurrent transients resulting from the two models are composites of the three component currents depicted by the blue, yellow and green arrows in Fig. 4. These three component transients are presented in Fig. 6. In the RMIL model, all three contribute to the decay of the initial cathodic current after turning on the light (Fig. 6a, b), whereas the spike of anodic current after turning off the light comes mostly from TMPD oxidation by the FTO. Simulation of the shapes of both spikes was achieved by optimization of the rate constants of P^+^ reduction by TMPD and Q_B_^−^ oxidation by TMPD^+^ (${k_{{\text{TMPD}} - {\text{P}}}}$ and ${k_{{\text{TMPD}} - {\text{Q}}}}$ in Fig. 4b) and simulation of their amplitudes by optimization of the rate constants of the TMPD/TPMD^+^–FTO interaction (${k_{{\text{TMPD}} - {\text{FTO}}}}$) and TMPD/TMPD^+^ diffusion (${k_{{\text{diff}}}}$). The region of steady photocurrent is dominated by opposing contributions from the primary donor P^+^ and P^T^ states interaction with TiO_2_ (yellow and blue in Fig. 6a, b), and the correct sign and amplitude of the current in this region was obtained by optimizing the corresponding rate constants ${k_{{\text{dirP}}}}$ and ${k_{{\text{dirT}}}}$.

In the IP model, the cathodic spike after turning on the light comes mostly from reduction of P^+^ by the TiO_2_ (Fig. 4a, yellow arrows and Fig. 6c, d yellow line), whereas the anodic peak after turning off the light comes, as in RMIL model, from TMPD oxidation by the FTO. In this case, the rate constants for diffusion and the reaction between TMPD/TMPD^+^ and FTO were again optimized to simulate the shape of the current spikes. Furthermore, in the IP model for electrodes without TiCl_4_ treatment, the current from the TMPD–FTO interaction made a small contribution to the steady current (Fig. 6c), and thus had to be taken into account during the fitting procedure.

Irrespective of the model, the data in Fig. 6 demonstrate that competition between cathodic and anodic currents produces a low net output, the sign of which is sensitive to the relative amplitudes of the two currents.

From the values of parameters summarized in Table 1, one can calculate lifetimes of respective reactions as the reciprocals of rates for first-order reactions or the reciprocals of the product of rate constants and TMPD or TMPD^+^ concentration in the steady photocurrent phase (for second-order reactions) (Table 2). One can see that, for the RMIL model, direct electron transfer from P^T^ to TiO_2_ (${\tau _{{\text{dirT}}}}=20~{\text{ms}}$) is three orders of magnitude slower than the lifetime for P^T^ recombination (${\tau _{{\text{recT}}}}=50~\mu {\text{s}}$). A similar situation is found for electron transfer from TiO_2_ to P^+^ (${\tau _{{\text{dirP}}}}=10/22~{\text{s}}$) and recombination of the P^+^Q^−^ state (${\tau _{{\text{recPQ}}}}=100~{\text{ms}}$), with the former reaction being two orders of magnitude slower than the latter one. Hence, recombination processes that are much faster than direct electron transfer reactions between RCs and TiO_2_ are the most important factors underlying the overall low photocurrent efficiency (IPCE ≈ 10^−5^; Fig. 3) in the RMIL model. Other factors are a low efficiency of light capture (up to ~ 10%), and compensation between opposing cathodic and anodic currents. Artificially increasing the yield of triplet formation from 15 to 100% in the model increased both the cathodic and anodic current contributions, but did not significantly change the net current (data not shown). An additional factor responsible for the low IPCE of the cathodic current is donation of electrons by TMPD to P^+^ in the steady photocurrent region (${\tau _{{\text{TMPD}} - {\text{P}}0}}=3.8/4.0~{\text{s}}$) which is ~ 3–5 times faster than electron transfer from TiO_2_ to P^+^ (${\tau _{{\text{dirP}}}}=10/22~{\text{s}}$) and so short circuits the RC.

**Table 2:** Modelled lifetimes of the electron transfer reactions to and from RCs and the lifetimes of recombination reactions inside RCs in the two models

Parameter	Unit	Value			
		RMIL model no TiCl4	RMIL model with TiCl4	IP model no TiCl4	IP model with TiCl4
		(χ = 0)		(χ = 0.9)	
${\tau _{{\text{TMPD}} - {\text{P}}}}_{0}$	s	3.8	4.0	6.0 × 10^−3^	1.1 × 10^−2^
${\tau _{{\text{TMPD}} - {\text{Q}}}}_{0}$	s	13.9	13.3	6.1	1.8
${\tau _{{\text{dirT}}}}$	s	2 × 10^−2^		1.3 × 10^−4^	
${\tau _{{\text{dirP}}}}$	s	10.3	22.2	1.3 × 10^−4^	2.9 × 10^−3^
${\tau _{{\text{recT}}}}$	s	5 × 10^−5^			
${\tau _{{\text{recPQ}}}}$	s	1 × 10^−1^			

The first two lifetimes were calculated as *τ*_*TMPD*−*P0*_ = 1/(*k*_*TMPD*−*P*_ [TMPD]) and *τ*_*TMPD*−*Q0*_ = 1/(*k*_*TMPD*−*Q*_ [TMPD^+^]), where values for rate constants were taken from Table 1, whereas [TMPD] and [TMPD^+^] values were taken from 299 s of simulation (end of photocurrent, see Figs. S7 and S8), hence these values are reliable only for the stable photocurrent region. The remaining lifetimes were simply calculated as reciprocals of corresponding rate constants shown in Table 1 (${\tau _{{\text{dirT}}}},~{\tau _{{\text{dirP}}}}$) or taken from literature (${\tau _{{\text{recT}}}},{\tau _{{\text{recPQ}}}}$) (Blankenship et al. 1995; Frank et al. 1996)

In the IP model, the lifetimes for direct electron transfer between the TiO_2_ and P^+^ are much shorter than those in the RMIL model (6/11 ms c.f. 3.8/4.0 s), and either comparable with wasteful recombination reactions (compare with the values of ${\tau _{{\text{dirT}}}}$ and ${\tau _{{\text{recT}}}}$) or even shorter than that (compare with the values of ${\tau _{{\text{dirP}}}}$ and ${\tau _{{\text{recPQ}}}}$). Thus, in the IP model, the main factors responsible for the low overall current are no longer the competing recombination reactions, but instead, the compensating effect of the cathodic and anodic currents which diminishes the net current (of the order of ~ 100 nA) by about two orders of magnitude relative to the individual cathodic and anodic components (~ 10 µA each; compare Figs. 5, 6). This compensation is a consequence of a short circuit in electron transfer that can be summarized as ${{\text{P}}^{\text{T}}} \to {\text{Ti}}{{\text{O}}_2} \to {{\text{P}}^+}$. The other factors responsible for the low IPCE in the scenario are a small pool of active RCs (10%) and, as in the RMIL model, a low efficiency of capturing the light (up to ~ 10%). Also as in the RMIL model, an increase of yield of triplet formation up to 100% would not lead to significant change of the net current due to compensation between the current contributions.

To sum up, the models reveal four possible reasons, other than low absorbance, for the relatively low photocurrent output of the cell. They are (1) electron transfer rates between RCs and TiO_2_ lower than the rate of charge recombination within RCs (dominates in the RMIL model); (2) competition between anodic and cathodic photocurrents (dominates in the IP model); (3) a pool of photoelectrochemically inactive RCs (IP model only); (4) short-circuiting of the RCs by TMPD acting as both oxidant and reductant (especially in RMIL model).

For efficient DSSCs, typical lifetimes for electron injection into TiO_2_ by the photoexcited dye are of the order of 10^−10^ s (Martín et al. 2016) which is several orders of magnitude faster than the values obtained in this work for electron donation from P^T^. The most probable reason for this is the lack of an excess of energy for the state injecting the electron relative to the conduction band edge of the TiO_2_; as can be seen from Fig. 4, the triplet state of the RC primary donor is almost isoenergetic with the edge of the conduction band of TiO_2_. A possible way of improving this pathway would be to change the energy of the TiO_2_ conduction band through the addition of lithium ions (Yu et al. 2010) or the use of an alternative semiconductor such as ZnO with different energy levels. On the other hand, surface states are known to be low efficiency in terms of electron transfer (Frank and Bard 1975) and this pathway would be hard to improve.

Each of the two models presented in Fig. 5 seemed to be able to fit the experimental data well only in some parts of the time range, and it is possible that combination of these into a single, more complex model could lead to a better agreement between the experimental data and the simulation in all respects. Furthermore, in the existing models, there are five or six parameters that are chosen arbitrarily and may compensate each other, and it would be very useful to measure at least some of these in independent experiments. Although at this stage it is hard to clearly say which assumptions are proper for the studied system, the results obtained show that the proposed approach for modelling can give useful information about the operation mechanism of such a biohybrid photochemical device. The proposed model could be used to simulate data obtained by laboratories that have reported higher efficiencies of systems in which proteins and TiO_2_ have been combined (Lukashev et al. 2007; Mershin et al. 2012; Gizzie et al. 2015a; Kavadiya et al. 2016), to diagnose what could be improved to obtain even higher efficiencies.

## Conclusions

Our measured electrochemical and photoelectrochemical data, and the associated kinetic model, have produced new insights into the photocurrent output of photovoltaic cells fabricated from photosynthetic RCs and TiO_2_. The net observable photocurrent is proposed to consist of three parallel sources: (1) injection of electrons from the triplet state of P (anodic), (2) reduction of P^+^ by TiO_2_ (cathodic) and (3) oxidation/reduction of TMPD/TMPD^+^ by the FTO glass substrate (producing cathodic and anodic peaks). These combine to yield a relatively modest stable photocurrent of up to 300 nA cm^−2^ with an IPCE of up to 1.5 × 10^−3^%. The two models show two alternative main reasons for the low efficiency of the system, relatively fast inner recombination in the RMIL model and efficient recombination via TiO_2_ in the IP model. Deconstruction of the net current using the kinetic model provides insight into how the photocurrent amplitude may be enhanced in either a cathodic or anodic direction through future manipulation of the system. The efficiency of the system could not be improved significantly by changing only one parameter in the system, as any change influences both cathodic and anodic contributions to the current, which then compensate each other. There is therefore a need to both suppress one of the current contributions and improve the efficiency of the other.

## Experimental section

### Biological material

The *Rba. sphaeroides* RC used in this work was modified at the C-terminus of the PufM polypeptide with the sequence LALVPRGSSA**AHKKPSKSA**SA*HHHHHHHHHH* (see Sect. 1 of Supporting Information), using the same approach as described previously for His tag modification (Swainsbury et al. 2014). The synthetic DNA sequence used to prepare this construct is included in the Supporting Information. This sequence comprised a thrombin cleavage site (underlined), followed by an LSTB1 tag (Chen et al. 2009) to target binding to TiO_2_ (bold), followed by a deca-histidine tag to facilitate purification (italics). The addition of the histidine tag also ensured the whole population of purified RCs which contained the LSBT1 tag by selecting for proteins that had not had the tag cleaved during protein assembly or protein purification. The modified RC gene was expressed in *Rba. sphaeroides* strain DD13, producing an antenna-deficient transconjugant strain with the modified RC as the sole pigment-protein (Swainsbury et al. 2014). This strain was grown in the dark, and RCs purified by a combination of nickel affinity chromatography and size exclusion chromatography, as described elsewhere (Swainsbury et al. 2014).

### Preparation of TiO_2_ paste

TiO_2_ paste for photocurrent measurements was prepared by applying a procedure based on the one described by Woronowicz et al. (2012) to 50 nm anatase nanoparticles (MKnano, 98% pure). Briefly, TiO_2_ nanoparticles were mixed with double-distilled water with acetylacetone followed by slow addition of double-distilled water with Triton X-100. Electrodes prepared using this procedure were denoted W-50.

TiO_2_ paste for absorption measurements and IPCE was prepared by a procedure based on the one described by Ito et al. (Ito et al. 2007), with the exception that a three-roller mill was not used. It was chosen for absorption measurements due to its lower light scattering and similar photocurrent results to W-50 (data not shown). Briefly, nanoparticles were mixed with water, acetic acid, ethanol, terpineol and ethyl cellulose by subsequent treatments with a mortar, magnetic stirrer and ultrasonic horn (Sonics Vibra-Cell VCX130). Excess ethanol was evaporated using a rotary evaporator. Ethanol and acetic acid were from Avantor, and all other chemicals were from Sigma-Aldrich. Electrodes prepared using this procedure were denoted I-50.

### Assembly of working electrodes

Glass slides covered with FTO (Sigma-Aldrich, TEC 15) were washed in an ultrasonic bath (CT-Brand CT-432H1) sequentially in water with dish soap, double-distilled water and ethanol for 10 min each. TiO_2_ paste was then deposited on the cleaned FTO glass using a doctor-blading technique (for paste formulation see above) using Scotch 3M Magic Tape as a mask and to define layer thickness. This was followed by sintering in a Nabertherm 5/11 – P330 oven that was warmed up to 570 °C over 25 min and held at that temperature for a further 30 min. The active area of the TiO_2_ film was 0.25 cm^2^. After cooling to room temperature, 1 µL of a stock solution of ~ 230 µM RC protein in 20 mM Tris–HCl (pH 8.0)/0.1% LDAO (*N,N*-dimethyldodecylamine *N*-oxide) was drop casted onto the sintered substrate and left to dry at 4 °C in the dark overnight (the RC concentration was determined using an extinction coefficient of 288 mM^−1^ cm^−1^ for the RC absorbance band at 803 nm) (Straley et al. 1973). Coated films were then rinsed with 20 mM Tris–HCl (pH 8.0) to remove any loosely bound RCs, and dried under a flow of compressed air for around 10 s. Uncoated areas of the FTO glass were covered with Scotch 3M Magic Tape to prevent direct contact of mediator with the conductive surface and to reduce the dark current.

For some working electrodes (those denoted W-50-Cl), an additional treatment with TiCl_4_ was applied before deposition of the RCs on the TiO_2_ layer, as described previously (Sommeling et al. 2006). Briefly, after sintering as described above, the electrodes were immersed in a 50 mM TiCl_4_ (Sigma-Aldrich) aqueous solution for 30 min at 70 °C, followed by rinsing with double-distilled water and sintering again at 570 °C for 30 min. The aim of the TiCl_4_ treatment was to cover the mesoporous TiO_2_ structure, and any bare areas of FTO glass, with an additional thin layer of TiO_2_ (Sommeling et al. 2006).

### Characterization of working electrodes

Photochronoamperometry was conducted using PGSTAT204 Autolab potentiostat and an 861 nm LED (LED870-66-60, Roithner LaserTechnik GmbH – for spectrum see Figure S9). The intensity of light used was 29.3 ± 1.5 mW cm^− 2^, unless indicated differently. A home-made 3-D printed electrochemical cell (with a 1 × 5 × 4.5 cm (L × W × H) glass cuvette for the electrolyte compartment) was used for all electrochemical experiments in a three-electrode configuration. The reference electrode was Ag/AgCl with 3 M KCl (+ 210 mV vs. SHE—standard hydrogen electrode) and the counter electrode was a Pt wire. The electrolyte solution was 250 µM TMPD (*N,N,N′,N′*-tetramethyl-*p*-phenylenediamine; Sigma-Aldrich) in 20 mM Tris–HCl (pH 8.0). All constant-potential electrochemical measurements were conducted at + 225 mV versus SHE as this was the average open-circuit potential (OCP) in the dark.

Action spectra were recorded using a PGSTAT302N Autolab potentiostat coupled with a photoelectric spectrometer (Instytut Fotonowy). For each wavelength (in 10 nm steps), the light was turned on for 10 s and off for 10 s while recording the current at an applied potential of + 225 mV versus SHE. For the photocurrent amplitude, the average value over the last 2 s of dark current was subtracted from the average value of the last 2 s of light current for each wavelength. The photocurrent amplitudes were then corrected for the intensity of the incident light.

Absorption spectra of TiO_2_ electrodes were measured using a Jasco V-770 spectrophotometer with an integrating sphere (ILN-925). Scanning electron microscopy (SEM) of TiO_2_ electrodes was performed using a Jeol 7001TTLS microscope with an acceleration voltage of 13 kV and current on sample of 35 pA. Samples were coated with thin layer of gold prior to SEM measurements in order to reduce surface charging.

## Electronic supplementary material

Below is the link to the electronic supplementary material.

Supplementary material 1 (PDF 1139 KB)
