# Supplementary material for: Modelling of the cathodic and anodic photocurrents from Rhodobacter sphaeroides reaction centres immobilized on titanium dioxide
Source: Photosynth Res. 2018 Jul 3;138(1):103–14. doi: 10.1007/s11120-018-0550-8 (PMC6208573; doi:10.1007/s11120-018-0550-8)
Supplement: Supplementary file 1 — Supplementary material 1 (PDF 1139 KB) [file 11120_2018_550_MOESM1_ESM.pdf]

Supporting Information

Modelling of the Cathodic and Anodic Photocurrents from *Rhodobacter sphaeroides* Reaction Centers Immobilized on Titanium Dioxide

Rafał Białek[1*], David J.K. Swainsbury[2†], Maciej Wiesner[1,3], Michael R. Jones[2] and Krzysztof Gibasiewicz[1*]

[1] Faculty of Physics, Adam Mickiewicz University in Poznań, ul. Umultowska 85, 61-614 Poznań, Poland
[2] School of Biochemistry, Biomedical Sciences Building, University of Bristol, University Walk, Bristol, BS8 1TD, United Kingdom
[3] NanoBioMedical Center, Adam Mickiewicz University in Poznań, ul. Umultowska 85, 61-614 Poznań, Poland
[†] Present address: Department of Molecular Biology and Biotechnology, University of Sheffield, Sheffield S10 2TN, United Kingdom
* e-mail: rafal.bialek@amu.edu.pl, krzyszgi@amu.edu.pl

SECTION 1 – DETAILS OF THROMBIN CLEAVABLE LSTB1-HIS10 TAG

Protein
LALVPRGSSA**AHKKPSKSA**SA_HHHHHHHHHH*_

DNA
CTGGTGCCGCGCGGGAGCAGCGCC**GCCCACAAGAAGCCGTCGAAGTCGGCC**AGCGCC_CATCACCACCATCAT_
_CACCATCACCACCAC_TGA

SECTION 2 – CHARACTERIZATION OF TiO_2 LAYERS

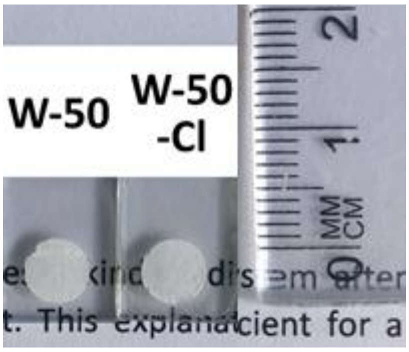

Figure S1. Images of TiO_2 layers before addition of RCs. The ruler is added for scale and text background to illustrate opacity.

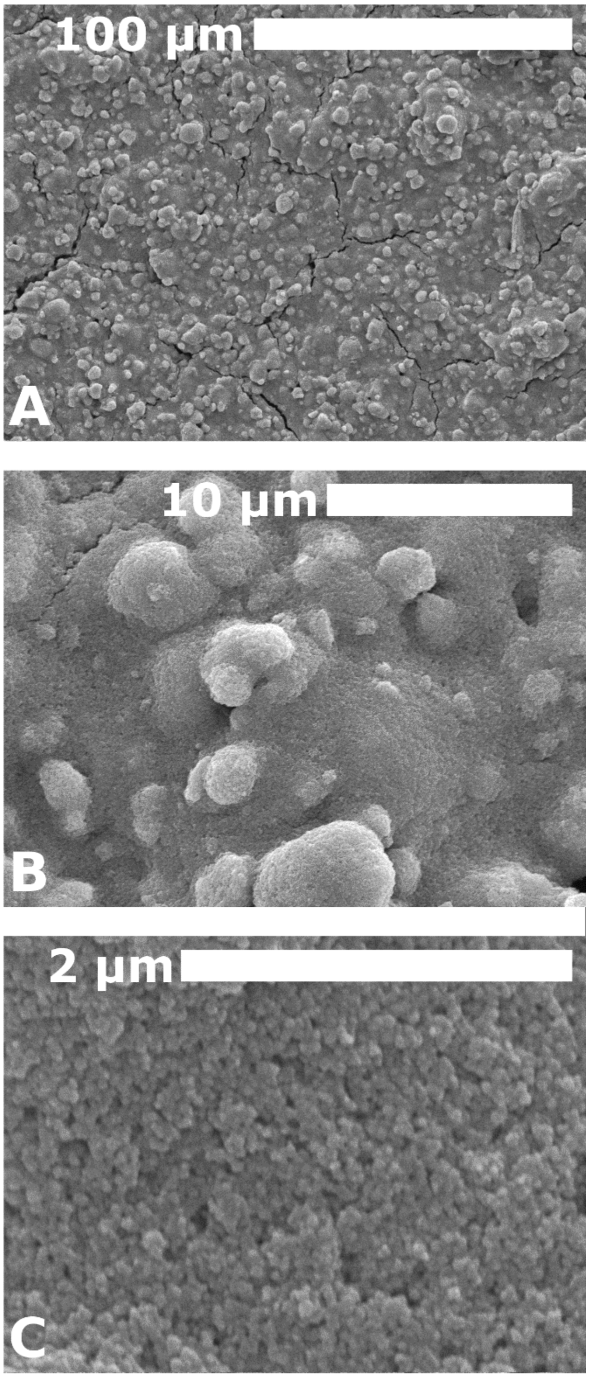

Figure S2. SEM micrographs of TiO_2 nanoparticle layers on FTO-glass after sintering and prior to protein deposition (no $TiCl_4$ treatment). Images show W-50 layers at different magnifications. All surfaces were covered with a thin layer of gold via sputtering to decrease surface charge and increase resolution.

SECTION 3 – TMPD ABSORPTION SPECTRUM

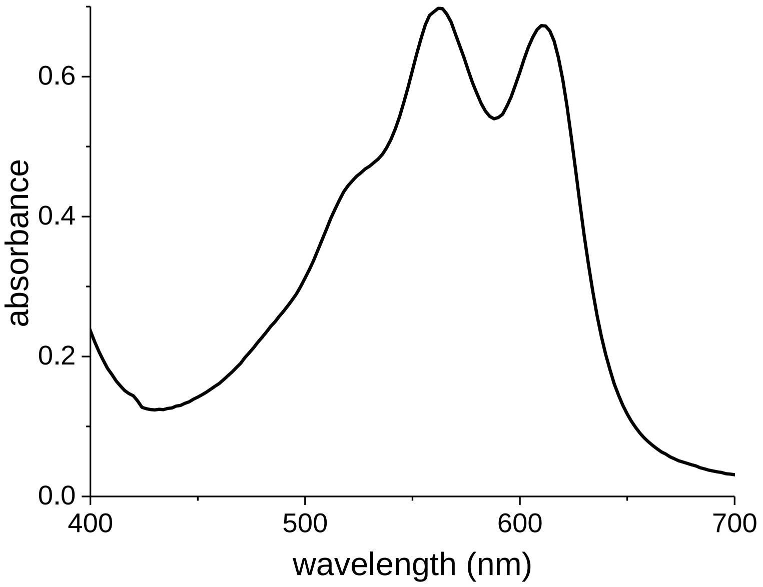

Figure S3. Absorption spectrum of 500 µM TMPD in 20 mM Tris-HCl (pH 8.0). The absorption bands originate from the oxidized form $TMPD^{+}$.

SECTION 4 – ABSORBANCE PROPERTIES OF BOUND RCS

Binding of RCs to the surface of deposited TiO_2 pastes caused some changes to the native absorbance spectrum of the bacteriochlorin cofactors (Figure S4A). The band around 865 nm attributable to the primary donor BChls was missing, an often-observed effect that can be attributed to oxidation of P in the air-dried sample (Moss et al. 1991). In addition, the 800-nm band attributable to the two accessory BChls was reduced in intensity relative to the 760-nm band attributable to the two RC BPhes. There are two possible reasons for such a change. The first one is a damage to the RC protein such that a BChl is detached from its native binding pocket – such a change would be expected to cause a reduction in the absorbance bands at 865 and/or 800 nm and an increase in absorbance around 760 nm due to the appearance of "free" BChl. The second is pheophytinization of a proportion of the RC BChls such that their central Mg^{2+} metal is replaced by two protons but they are retained in the protein scaffold in their binding pocket(s).

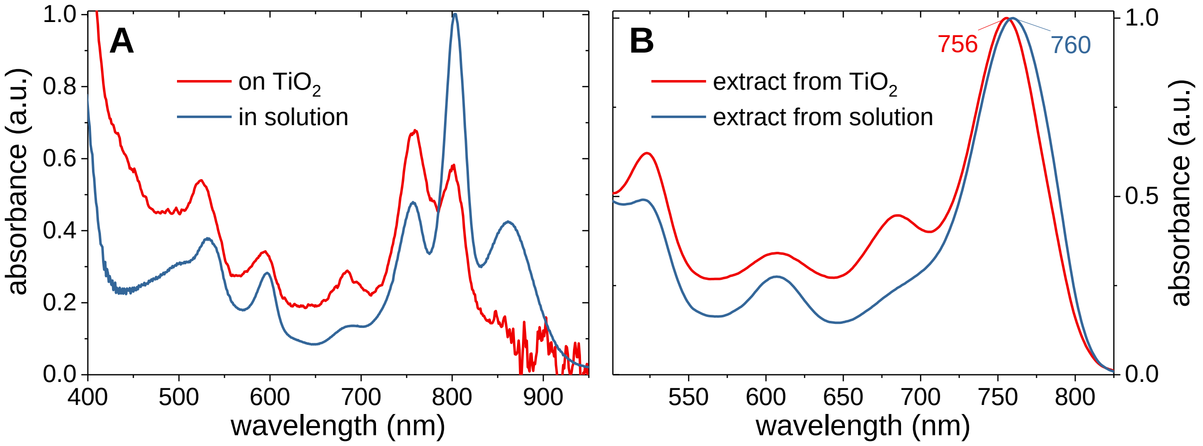

Figure S4. Absorbance properties revealing cofactor composition of RCs in solution and adhered to TiO_2. (A) Absorption spectrum of RCs immobilized on I-50 film partially corrected for TiO_2 scattering compared with the absorption spectrum of RCs in 20 mM Tris-HCl (pH 8.0)/0.1 % LDAO. The spectra were normalized to the same sum of concentrations of bacteriopheophytins and bacteriochlorophylls. (B) Comparison of absorption spectra of methanolic extracts of RCs in solution and immobilized on TiO_2. Absorbance maxima are indicated.

To investigate this change, bacteriochlorin pigments were extracted using methanol from RCs in solution and immobilized on TiO_2. For extraction of pigments with methanol (Avantor) a 2 µL aliquot of RC stock solution or a RC-coated TiO2 slide was immersed in 500 µL methanol and vigorously mixed for 1 min. The resulting solution was centrifuged at 12,100 g and the absorbance spectrum of the supernatant recorded using a Hitachi U-2800A spectrophotometer. The absorbance spectrum of pigments extracted from RCs immobilized on TiO_2 showed a blue-shift of the longest-wavelength absorbance band relative to that of pigments extracted from RCs in solution (Figure 2B). This spectral change is characteristic of a higher amount of BPhe relative to BChl, as the absorption maximum of BPhe in methanol is blue-shifted relative to that of BChl (Straley and Clayton 1973). This indicated that pheophytinization and not dissociation of BChl was responsible for the spectral changes shown in Figure 2A. In addition a band appeared at 680 nm in the spectrum of the TiO_2 RC extract that was probably attributable to a small amount of BChl decomposition products other than BPhe, such as 3-acetyl chlorophyll (Clayton 1966; Makhneva et al. 2016). With the assumption that a BPhe in a binding pocket normally occupied by a monomeric BChl has the same absorption spectrum as a BPhe in its native binding pocket, the spectrum of RCs deposited on TiO_2 in Figure 2A was normalized to the same total number of four BPhe and monomeric BChl molecules as for native RCs in solution.

This approach allowed estimation of a ratio of BPhe:monomeric-BChl of 2.8:1.2 in the TiO_2-bound RCs, which means that an average of 0.8 monomeric BChls per RC had undergone pheophytinization (see next section for a full account of the derivation of these values and the normalization method).

Further evidence that the spectral change undergone by RCs on binding to the TiO_2 electrode was due to *in situ* pheophytinization came from the similarity the ratio of amplitudes of the bands at 760 nm and 800 nm between the IPCE action spectrum for an I-50 electrode and the absorbance spectrum of the electrode. As free BChl pigments released from their binding pockets due to photodamage would not be expected to contribute to a cathodic photocurrent (Tsui et al. 2014), this matching supports the conclusion that RCs on the electrode surface had undergone some conversion of monomeric BChls to BPhe, producing the observed absorbance change. Furthermore, it suggested that the BChl being pheophytinized was B_B (see inset in Figure 1), as it does not take part in the electron transfer process (Kamran et al. 2015). A pheophytinization of B_A would be expected to suppress electron transfer in RC. The vulnerability of B_B to pheophytinization could be related to the fact that it takes part in photoprotection against triplet states, so there is a bigger risk for it to be damaged (Frank et al. 1996). A final point to note is that the IPCE action spectrum had a band at 865 nm attributable to the P BChls, supporting the conclusion that this band is bleached due to P oxidation when RCs are adhered to the TiO_2 electrode in air, but this bleaching is reversible after immersion in a solution of suitable redox potential.

SECTION 5 – ANALYSIS OF PHEOPHYTINIZATION

For normalization of absorption spectra of RCs bound to the surface of TiO_2 the following assumptions were made: (1) absorbance at 800 nm comes only from the accessory BChls, while that at 760 nm comes only from BPhe; (2) the only change in spectrum comes from transformation of BChl into BPhe; (3) the extinction coefficient of BPhe is the same for both natural positions of BPhe in the RC (i.e. H_A and H_B) and for BPhe at a location normally occupied by a monomeric BChl (i.e. BPhe formed after pheophytinization of B_A or B_B). These assumptions lead to the following set of equations:

$$\frac{A_{760(sol)}}{\varepsilon_{BPhe}}+\frac{A_{800(sol)}}{\varepsilon_{BChl}}=\frac{A_{760(TiO_2)-normalized}}{\varepsilon_{BPhe}}+\frac{a\cdot A_{760(TiO_2)-normalized}}{\varepsilon_{BChl}} \tag{S1}$$

$$a=\frac{A_{800(TiO_2)-measured}}{A_{760(TiO_2)-measured}} \tag{S2}$$

$$\varepsilon_{BPhe}=\frac{A_{760(sol)}}{2} \tag{S3}$$

$$\varepsilon_{BChl}=\frac{A_{800(sol)}}{2} \tag{S4}$$

Where: $A_{xxx(sol)}$ is the absorbance at *xxx* nm for RCs in solution, $A_{xxx(TiO_2)-normalized}$ is the absorbance at *xxx* nm for RCs on TiO_2 after normalization, $A_{xxx(TiO_2)-measured}$ is the absorbance at *xxx* nm for RCs on TiO_2 directly from measurements, ε_{yyy} is a dimensionless quantity proportional to the extinction coefficient for species *yyy*, and a is the experimental ratio of measured absorbances. Equations (S3) and (S4) take into account that there are normally two BPhes and two accessory BChls per RC molecule.

After solving equations S1 to S4, one obtains:

$$A_{760(TiO_2)-normalized}=\frac{2\cdot A_{760(sol)}\cdot A_{800(sol)}}{a\cdot A_{760(sol)}+A_{800(sol)}} \tag{S5}$$

The absorbance spectrum of RCs on TiO_2 presented in Figure 2 was normalized to this value at the maximum around 760 nm. The normalized absorption spectrum was then used to calculate an average number (*n*) of BPhes and accessory BChls per RC molecule using the equations:

$$n_{BChl}=\frac{A_{800(TiO_2)-normalized}}{\varepsilon_{BChl}} \tag{S6}$$

$$n_{BPhe}=\frac{A_{760(TiO_2)-normalized}}{\varepsilon_{BPhe}} \tag{S7}$$

SECTION 6 – ELECTROCHEMICAL PROPERTIES OF THE ELECTROLYTE

The supporting electrolyte was 20 mM Tris-HCl (pH 8.0). TMPD, which has previously been used as a component of solar cells based on *Rba. sphaeroides* RCs (Tan et al. 2012; Ravi et al. 2017), has two steps of oxidation (Figure S5). However only the first occurring at a formal potential of +260 mV vs SHE is of use because the doubly oxidized form, present at potentials over 700 mV vs SHE, undergoes decomposition with displacement of dimethylamine (Brownson and Banks 2014). The contribution of particular redox states to the electroactive species can be calculated by analysis of the values of stable currents in cyclic voltammetry (CV) scans on the right and left side of the formal redox potential (Figure S5), as these currents depend on the bulk concentrations of either reduced or oxidized species (assuming similar diffusion coefficients for reduced and oxidized form) (Zoski 2007). In the 1.2 mM solution the TMPD (neutral) and $TMPD^{+}$ (monocationic) forms dominated in a ~1:1 ratio (see lengths of A and B line segments in Figure S5 and next section for the derivation of the method; the diffusion coefficients of TMPD and $TMPD^{+}$ do not differ by more than 15 %) (Wang et al. 1997). The OCP of a freshly prepared solution of TMPD oscillated around +225 mV vs SHE, and so this potential was applied in all subsequent photocurrent measurements in order to minimize the dark current. It is also visible as the potential near which the CV curve in Figure S5 crosses zero current line.

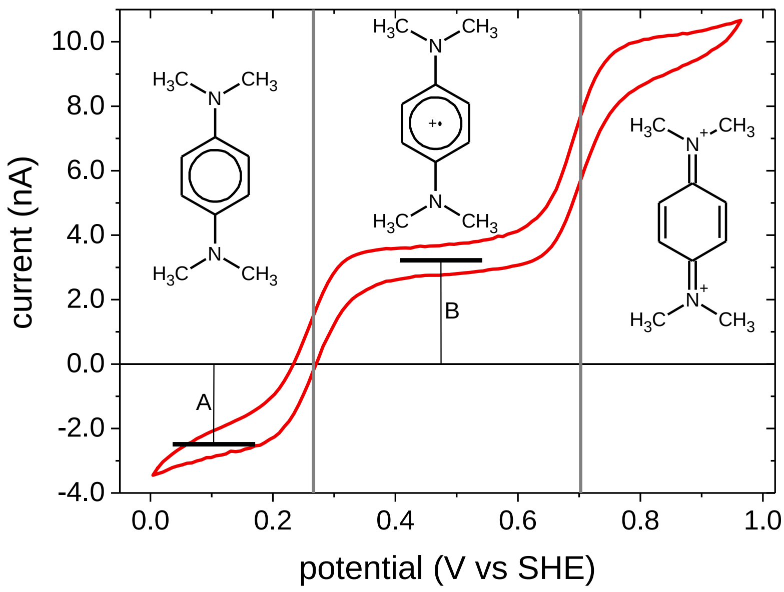

Figure S5. Electrochemical properties of the electrolyte. Cyclic voltammogram of 1.2 mM TMPD in 20 mM Tris-HCl (pH 8.0) on a 25 µm platinum disc microelectrode at a scan rate of 100 mV s^{-1}. The structures of the three redox forms of TMPD (neutral, mono- and bicathionic) are presented. In this and similar solution used for photocurrent measurements the bulk concentrations of the neutral and monocationic forms are similar, as estimated from similar positive and negative currents at ~+480 mV (B) and ~+110 mV (A) potentials (vs SHE), respectively (see text for details). The two long vertical lines indicate the formal redox potentials of neutral/monocationic and monocationic/bicationic TMPD pairs at +260 mV and +700 mV, respectively.

SECTION 7 – DETERMINATION OF THE TMPD/TMPD+ RATIO

The steady-state current in cyclic voltammetry measurements on a microelectrode is given by (Bard and Faulkner 2001):

$$i_{ss} = 4nFDC^{*} \tag{S8}$$

Where: n is the number of electrons transferred within reaction, F is Faraday's constant, D is the diffusion coefficient of the reacting species (either the oxidized or reduced form), and C^{*} is the bulk concentration of the reacting species.

This equation is valid only for microelectrode, as it allows probing bulk concentration of species. It is because of the low current flow, which does not much affect the local concentration of species as well as diffusion around microelectrode is closer to the spherical one than that on the macroelectrode. Spherical diffusion ensures efficient mass transport of species to the surface of electrode from the bulk volume (Bard and Faulkner 2001).

Using values for the cathodic and anodic steady-state currents (lines A and B in Figure S5) one can determine the concentration ratio of the oxidized and reduced forms of the redox mediator from:

$$\frac{C_{ox}^{*}}{C_{red}^{*}} = \frac{i_{cath}}{i_{an}} \cdot \frac{D_{red}}{D_{ox}} \tag{S9}$$

SECTION 8 – DESCRIPTION OF KINETIC MODEL

Physical details

Figure S6 shows the processes and associated rate constants included in the model used to simulate experimental photocurrent data. For simplicity only the RC states involving the primary electron donor P and terminal quinone acceptor Q are considered due to the much shorter lifetimes of other RC states (Blankenship et al. 1995). It is assumed that only a fraction $(1-\chi)$ of RCs are fully functional meaning that they can efficiently conduct electron transfer between TiO_2 and the mediator. The remaining fraction (χ) can absorb light but undergo wasteful charge recombination and dissipate energy without contribution to the photocurrent, and therefore represent parasitic absorption. The number of photons absorbed per second per area unit is counted from the absorbance of the whole system (see Equations S19 and S20). The triplet state P^T can be formed only in RCs in a closed state (PQ^-) as a result of $P^+H_A^- \rightarrow P^TH_A$ charge recombination, with a quantum yield Φ_T that is used as a parameter.

Diffusion of TMPD is, for simplicity, simulated as the exchange of reduced and oxidized forms of the mediator near the working electrode (TMPD, $TMPD^+$) with the bulk volume ($TMPD_{bulk}$, $TMPD^+_{bulk}$) and is characterized by rate constant k_{diff}. Thus, the flux of diffusion is proportional to the concentration difference between bulk and the region in immediate proximity of the working electrode (see Equation S17), which in general doesn't have to be strictly correct, especially while the concentrations of species are changing rapidly. It is a place for future possible improvements of the model.

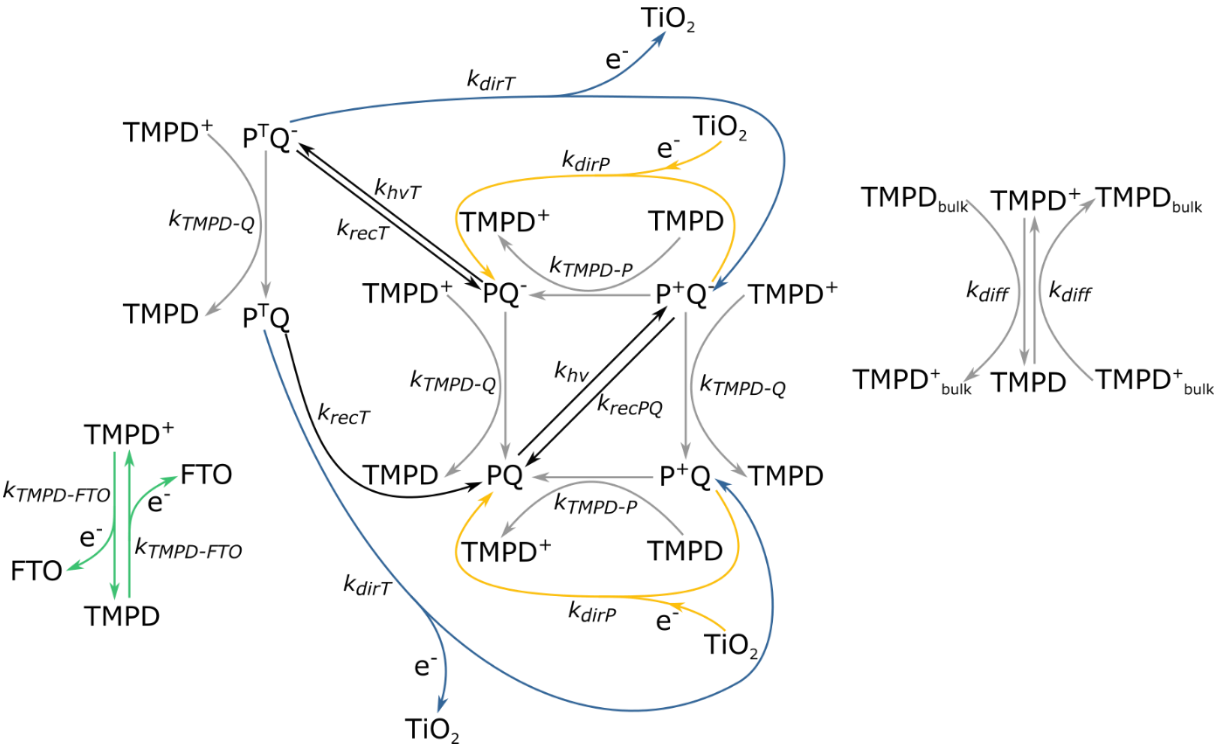

Figure S6. Schematic of the processes included in the kinetic model. The colors of arrows correspond to those indicating processes in Figure 5. Six RC states are considered: PQ, $P^+Q_A^-$, P^+Q_A, PQ_A^-, $P^TQ_A^-$, and P^TQ_A. Four of these states may exchange electrons with TMPD/$TMPD^+$: $P^+Q_A^-$, P^+Q_A, PQ_A^-, $P^TQ_A^-$. Two of the states may inject the electron to TiO_2: $P^TQ_A^-$ and P^TQ_A. Two of the states may accept an electron from TiO_2: $P^+Q_A^-$ and P^+Q_A. Two of the states may be excited by light: PQ_A and PQ_A^-.

Mathematical model

The differential equations presented below (S10 – S20) are the mathematical expression of the model presented in Figure S6. Most of the symbols are self-explanatory or are presented in Figure S6 (those with "n" at the beginning depict RCs inactive in photocurrent generation). The others are:

$[x]$ – concentration of state/species x,
$[TMPD_0]$ – initial concentration of TMPD (sum of reduced and oxidized species),
$[PQ_0]$ – total concentration of all RCs in the porous layer (equal to initial concentration of the RCs in state PQ assuming χ=0),
χ – fraction of RCs inactive in photocurrent generation,
l – thickness of the mesoporous TiO_2 layer,
ε – extinction coefficient of P at 861 nm,
P_0 – intensity of the incident light in units of power per unit area,
t – time,
t_{ON} – time of turning on the light,
t_{OFF} – time of turning off the light,
Φ_T – triplet formation quantum yield.

$$\frac{d[PQ]}{dt} = -k_{h\nu} + k_{recPQ}[P^+Q^-] + k_{TMPD-P}[P^+Q][TMPD] + k_{TMPD-Q}[PQ^-][TMPD^+] + k_{recT}[P^TQ] + k_{dirP}[P^+Q] \quad \text{(S10)}$$

$$\frac{d[P^+Q^-]}{dt} = k_{h\nu} - k_{recPQ}[P^+Q^-] - k_{TMPD-P}[P^+Q^-][TMPD] - k_{TMPD-Q}[P^+Q^-][TMPD^+] + k_{dirT}[P^TQ^-] - k_{dirP}[P^+Q^-] \quad \text{(S11)}$$

$$\frac{d[P^+Q]}{dt} = -k_{TMPD-P}[P^+Q][TMPD] + k_{TMPD-Q}[P^+Q^-][TMPD^+] + k_{dirT}[P^TQ] - k_{dirP}[P^+Q] \quad \text{(S12)}$$

$$\frac{d[PQ^-]}{dt} = k_{TMPD-P}[P^+Q^-][TMPD] - k_{TMPD-Q}[PQ^-][TMPD^+] - k_{h\nu T} + k_{recT}[P^TQ^-] + k_{dirP}[P^+Q^-] \quad \text{(S13)}$$

$$\frac{d[nPQ]}{dt} = 0 \quad \text{(S14)}$$

$$\frac{d[P^TQ]}{dt} = k_{TMPD-Q}[P^TQ^-][TMPD^+] - k_{recT}[P^TQ] - k_{dirT}[P^TQ] \quad \text{(S15)}$$

$$\frac{d[P^TQ^-]}{dt} = k_{h\nu T} - k_{TMPD-Q}[P^TQ^-][TMPD^+] - k_{recT}[P^TQ^-] - k_{dirT}[P^TQ^-] \quad \text{(S16)}$$

$$\frac{d[TMPD^+]}{dt} = \frac{k_{diff}[TMPD][TMPD_0]}{2} - \frac{k_{diff}[TMPD^+][TMPD_0]}{2} + \frac{k_{TMPD-FTO}}{l}[TMPD] - \frac{k_{TMPD-FTO}}{l}[TMPD^+] - k_{TMPD-Q}[P^TQ^-][TMPD^+] - k_{TMPD-Q}[PQ^-][TMPD^+] - k_{TMPD-Q}[P^+Q^-][TMPD^+] + k_{TMPD-P}[P^+Q^-][TMPD] + k_{TMPD-P}[P^+Q][TMPD] \quad \text{(S17)}$$

$$\frac{d[TMPD]}{dt} = -\frac{d[TMPD^+]}{dt} \tag{S18}$$

$$k_{h\nu} = \\ = \begin{cases} 0 & t < t_{ON} \\ \frac{[PQ]}{[PQ] + [PQ^-] + [nPQ]} \left(1 - 10^{-l\,\varepsilon\,([PQ]+[PQ^-]+[nPQ])}\right) \frac{P_0 \lambda}{chN_A l} & t \in \langle t_{ON}, t_{OFF} \rangle \\ 0 & t > t_{OFF} \end{cases} \tag{S19}$$

$$k_{h\nu T} = \\ = \begin{cases} 0 & t < t_{ON} \\ \Phi_T \frac{[PQ^-]}{[PQ] + [PQ^-] + [nPQ]} \left(1 - 10^{-l\,\varepsilon\,([PQ]+[PQ^-]+[nPQ])}\right) \frac{P_0 \lambda}{chN_A l} & t \in \langle t_{ON}, t_{OFF} \rangle \\ 0 & t > t_{OFF} \end{cases} \tag{S20}$$

The equations described above together with boundary conditions (equations S21-S29) were solved using Wolfram Mathematica NDSolve function with parameters: $MaxStepSize = 0.1$, $MaxSteps = \infty$ and $WorkingPrecision = 25$.

$$[PQ](0) = (1 - \chi)[PQ_0] \tag{S21}$$

$$[nPQ](0) = \chi[PQ_0] \tag{S22}$$

$$[P^+Q^-](0) = 0 \tag{S23}$$

$$[P^+Q](0) = 0 \tag{S24}$$

$$[PQ^-](0) = 0 \tag{S25}$$

$$[P^TQ](0) = 0 \tag{S26}$$

$$[P^TQ^-](0) = 0 \tag{S27}$$

$$[TMPD](0) = [TMPD_0]/2 \tag{S28}$$

$$[TMPD^+](0) = [TMPD_0]/2 \tag{S29}$$

The resulting current was calculated using Equation S30:

$$I(t) = I_{TMPD}(t) + I_{triplet}(t) + I_{surface\,states}(t) = \\ = F\,\{k_{TMPD-FTO}\,([TMPD] - [TMPD^+]) \\ + k_{dirT}\,l\,([P^TQ^-] + [P^TQ]) - k_{dirP}\,l\,([P^+Q] + [P^+Q^-])\} \tag{S30}$$

Model Parameters

Simulations of photocurrent transients for electrodes both treated with $TiCl_4$ and not treated with $TiCl_4$ were performed with the parameters presented in S31. The extinction coefficient is taken from literature (Straley et al. 1973). The thickness of the TiO_2 layer is the value typical for DSSCs (Ito et al. 2007). Recombination rate constants are taken from literature (Blankenship et al. 1995; Frank et al. 1996). Values of k_{TMPD-P} and k_{TMPD-Q} were taken from the literature for freely diffusing RCs with TMPD (Agalidis and Velthuys 1986). The concentration of RCs was calculated using the Beer-Lambert

law and the value of light absorption at the Q_y maximum. The concentration of TMPD and the light intensity were as used in experiments.

$$
\begin{aligned}
&F = 96485\, C\, mol^{-1} \\
&c = 3 \cdot 10^{8}\, m\, s^{-1} \\
&N_A = 6.02 \cdot 10^{23}\, mol^{-1} \\
&\varepsilon = 14400\, mol^{-1}\, m^{2} \\
&l = 4 \cdot 10^{-6}\, \mathrm{m} \\
&k_{recPQ} = 10\, s^{-1} \\
&k_{recT} = 20000\, s^{-1} \\
&k_{TMPD-P} = 800\, mol^{-1}\, m^{3}\, s^{-1} \\
&k_{TMPD-Q} = 4\, mol^{-1}\, m^{3}\, s^{-1} \\
&[TMPD_0] = 0.25\, mol\, m^{-3} \\
&[PQ_0] = 1.2\, mol\, m^{-3} \\
&P_0 = 300\, W\, m^{-2} \\
&\Phi_T = 0.15
\end{aligned}
\tag{S31}
$$

Two sets of values were used for the remaining parameters according to two models. The "inactive pool" (IP) model assumed that 90 % of RCs dissipate energy quickly and do not contribute to the photocurrent. The "RC-mediator interface limited" (RMIL) model assumed that 100 % of RCs undergo charge separation and do not dissipate energy, but the values of k_{TMPD-P} and k_{TMPD-Q} are different from those available in the literature due to immobilization of RCs on the TiO_2 surface which hinders access of the mediator to reduced and oxidized cofactors within protein. In the case of this second model the values of k_{TMPD-P} and k_{TMPD-Q} were optimized to obtain the best fit to the photocurrent traces. The values for these parameters are shown in Table 1 in the main text.

Time-dependence of species concentrations

The time-dependence of the concentrations of all species are shown in Figures S9 and S10. Interpretation of these plots is presented in the main text.

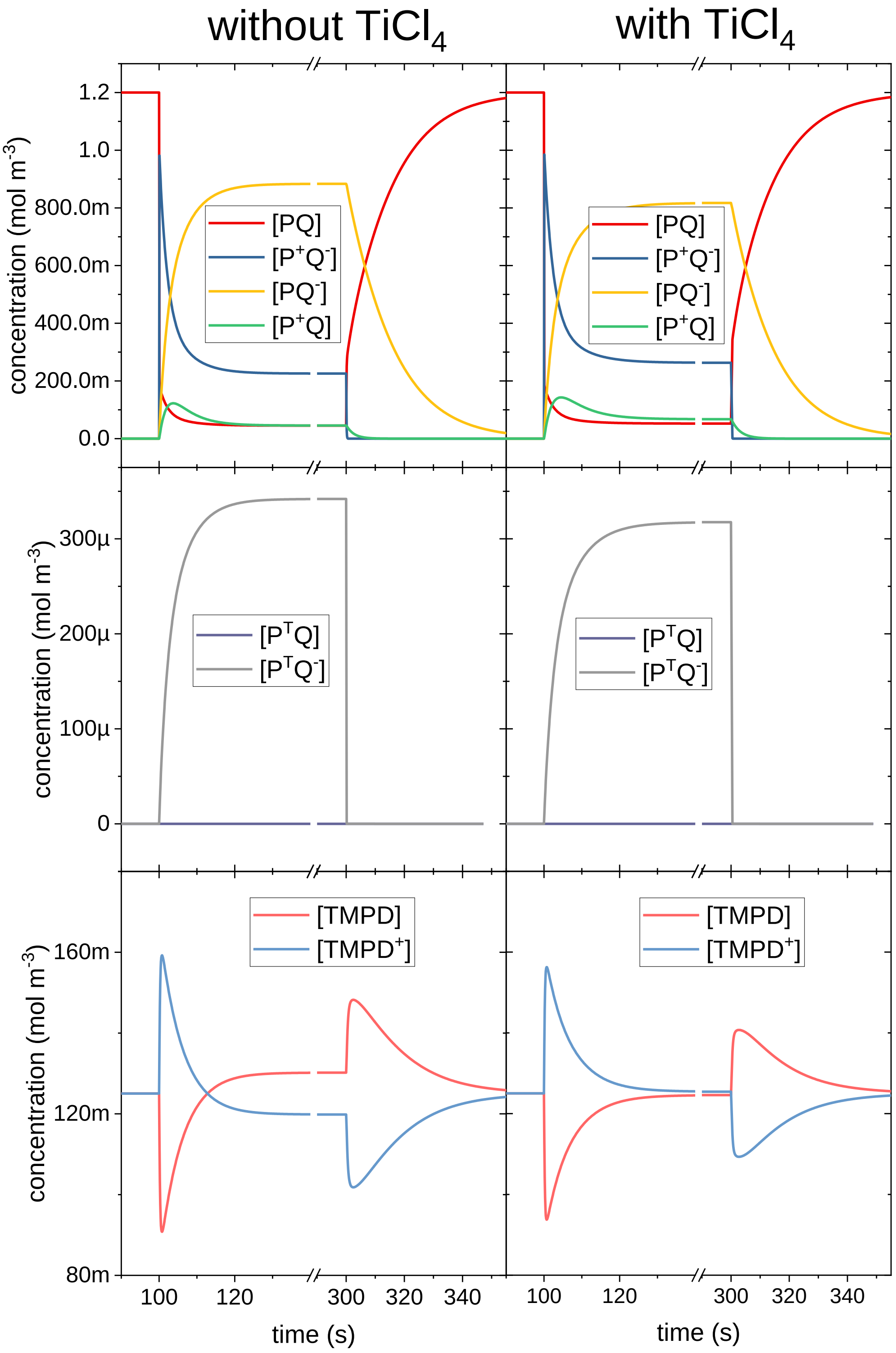

Figure S7. Species concentrations simulated using the RMIL model.

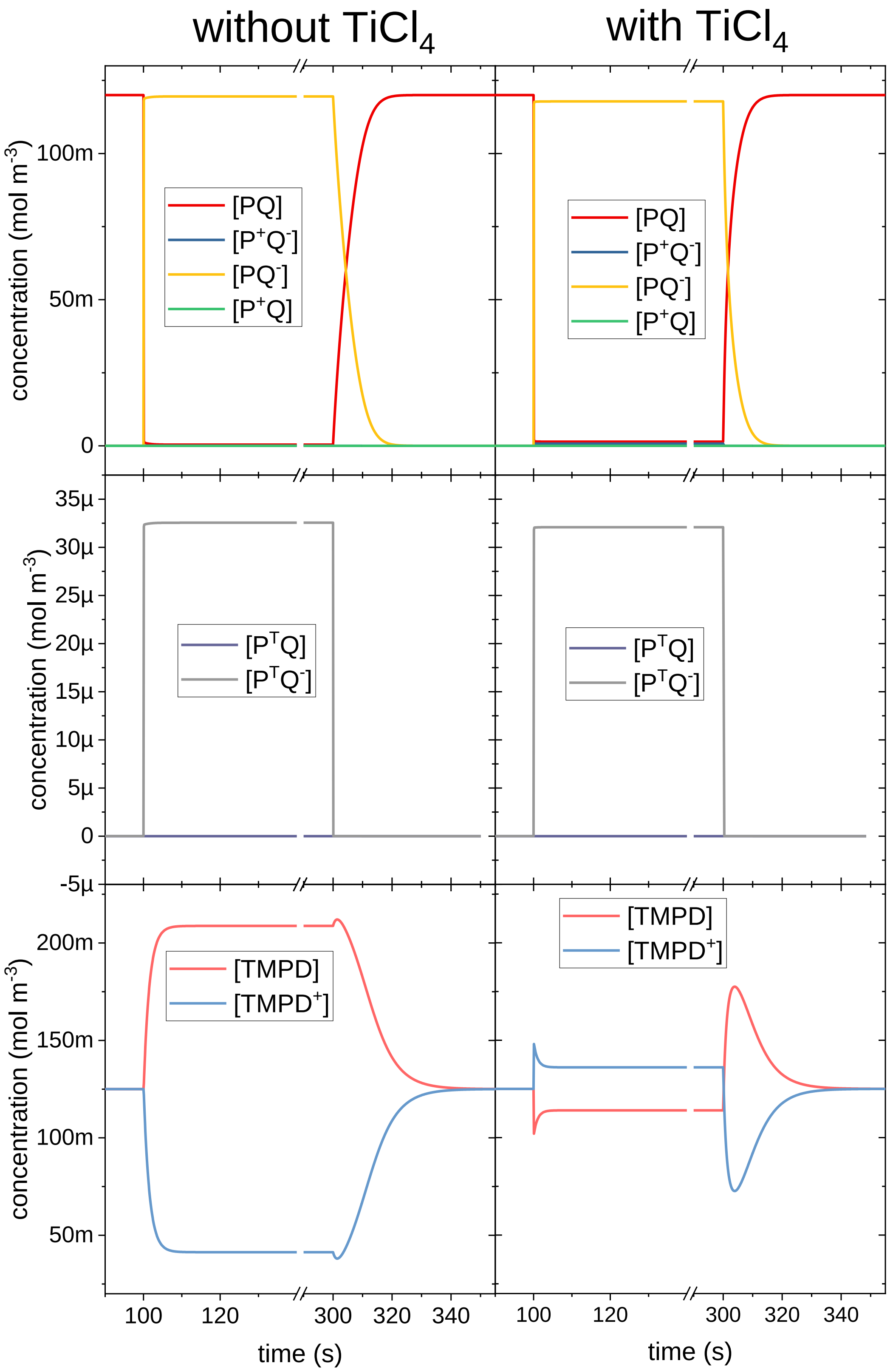

Figure S8. Species concentrations simulated using the IP model.

SECTION 9 – LED CHARACTERISTICS

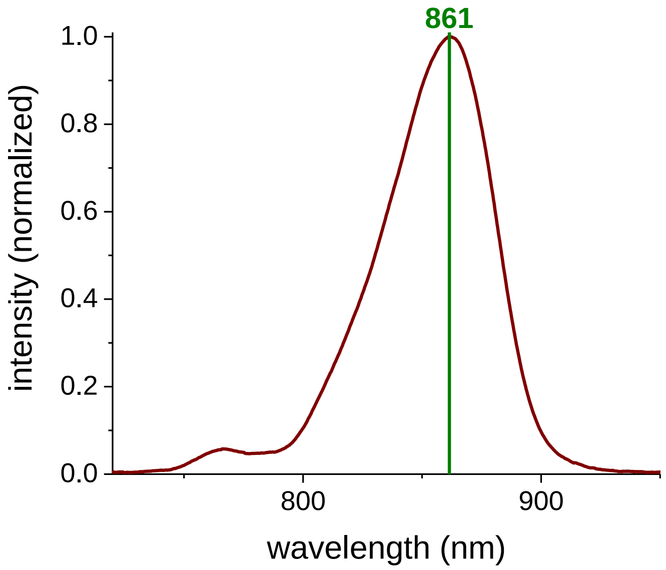

Figure S9. Spectrum of the LED used as the excitation light source for all experiments except action spectra. FWHM 53 nm, incident intensity 29.3 ± 1.5 mW cm^{-2}.

REFERENCES

Agalidis I, Velthuys BR (1986) Oxidation of Q_A^- and of Q_B^- of photosynthetic reaction centers by an artificial acceptor. FEBS Lett 197:263–266 . doi: 10.1016/0014-5793(86)80339-8

Bard AJ, Faulkner LR (2001) Electrochemical Methods: Fundamentals and Applications, 2nd editio. Wiley

Blankenship RE, Madigan MT, Bauer CE (eds) (1995) Anoxygenic Photosynthetic Bacteria. Kluwer Academic Publishers, Dordrecht

Brownson DAC, Banks CE (2014) Interpreting Electrochemistry. In: The Handbook of Graphene Electrochemistry. Springer London, London, pp 23–77

Clayton RK (1966) Spectroscopic Analysis of Bacteriochlorophylls in vitro and in vivo. Photochem Photobiol 5:669–677 . doi: 10.1111/j.1751-1097.1966.tb05813.x

Frank HA, Chynwat V, Posteraro A, et al (1996) Triplet state energy transfer between the primary donor and the carotenoid in *Rhodobacter sphaeroides* R-26.1 reaction centers exchanged with modified bacteriochlorophyll pigments and reconstituted with spheroidene. Photochem Photobiol 64:823–831 . doi: 10.1111/j.1751-1097.1996.tb01842.x

Ito S, Chen P, Comte P, et al (2007) Fabrication of screen-printing pastes from TiO_2 powders for dye-sensitised solar cells. Prog Photovoltaics Res Appl 15:603–612 . doi: 10.1002/pip.768

Kamran M, Friebe VM, Delgado JD, et al (2015) Demonstration of asymmetric electron conduction in pseudosymmetrical photosynthetic reaction centre proteins in an electrical circuit. Nat Commun 6:6530 . doi: 10.1038/ncomms7530

Makhneva ZK, Ashikhmin AA, Bolshakov MA, Moskalenko AA (2016) 3-Acetyl-chlorophyll formation in light-harvesting complexes of purple bacteria by chemical oxidation. Biochem 81:176–186 . doi: 10.1134/S0006297916020115

Moss DA, Leonhard M, Bauscher M, Mäntele W (1991) Electrochemical redox titration of cofactors in the reaction center from *Rhodobacter sphaeroides*. FEBS Lett 283:33–36 . doi: 10.1016/0014-5793(91)80547-G

Ravi SK, Yu Z, Swainsbury DJKK, et al (2017) Enhanced Output from Biohybrid Photoelectrochemical Transparent Tandem Cells Integrating Photosynthetic Proteins Genetically Modified for Expanded Solar Energy Harvesting. Adv Energy Mater 7:1601821 . doi: 10.1002/aenm.201601821

Straley SC, Clayton RK (1973) Extraction of oxidized bacteriochlorophyll from illuminated photosynthetic reaction center particles. Biochim Biophys Acta - Bioenerg 292:685–691 . doi: 10.1016/0005-2728(73)90016-9

Straley SC, Parson WW, Mauzerall DC, Clayton RK (1973) Pigment content and molar extinction coefficients of photochemical reaction centers from *Rhodopseudomonas spheroides*. Biochim Biophys Acta - Bioenerg 305:597–609 . doi: 10.1016/0005-2728(73)90079-0

Tan SC, Crouch LI, Jones MR, Welland M (2012) Generation of Alternating Current in Response to Discontinuous Illumination by Photoelectrochemical Cells Based on Photosynthetic Proteins. Angew Chemie Int Ed 51:6667–6671 . doi: 10.1002/anie.201200466

Tsui L, Huang J, Sabat M, Zangari G (2014) Visible Light Sensitization of TiO_2 Nanotubes by Bacteriochlorophyll-C Dyes for Photoelectrochemical Solar Cells. ACS Sustain Chem Eng 2:2097–2101 . doi: 10.1021/sc500386g

Wang RL, Tam KY, Compton RG (1997) Applications of the channel flow cell for UV-visible spectroelectrochemical studies Part 3. Do radical cations and anions have similar diffusion coefficients to their neutral parent molecules? J Electroanal Chem 434:105–114 . doi: 10.1016/S0022-0728(97)00114-9

Zoski CG (ed) (2007) Handbook of Electrochemistry. Elsevier, Amsterdam
